# Genome‐Wide Association Study of Pain in Parkinson's Disease Implicates *TRPM8* as a Risk Factor

**DOI:** 10.1002/mds.28001

**Published:** 2020-02-20

**Authors:** Nigel M. Williams, Leon Hubbard, Cynthia Sandor, Caleb Webber, Hannah Hendry, Michael Lawton, Camille Carroll, K. Ray Chaudhuri, Huw Morris, Michele T. Hu, Donald G. Grosset, Christopher Kobylecki, Monty Silverdale, the UK Parkinson's Pain Study

**Affiliations:** ^1^ Division of Psychological Medicine and Clinical Neurosciences Cardiff University Cardiff United Kingdom; ^2^ Division of Neurology, Nuffield Department of Clinical Neurosciences Oxford University Oxford United Kingdom; ^3^ Department of Population Health Sciences, Bristol Medical School University of Bristol Bristol United Kingdom; ^4^ University of Plymouth and University Hospitals Plymouth National Health Service Trust Plymouth United Kingdom; ^5^ Department Basic and Clinical Neuroscience, The Maurice Wohl Clinical Neuroscience Institute King's College and King's College Hospital London United Kingdom; ^6^ Department of Clinical Neuroscience, University College London Institute of Neurology London United Kingdom; ^7^ Department of Neurology, Institute of Neurological Sciences Queen Elizabeth University Hospital Glasgow United Kingdom; ^8^ Department of Neurology, Salford Royal NHS Foundation Trust, Manchester Academic Health Science Centre University of Manchester Manchester United Kingdom

Chronic pain affects 60% to 85% of people with Parkinson's disease (PD) and has a strong negative effect on quality of life.^1^ Genetic factors are significantly associated with a variety of chronic pain conditions.^2^ Identifying additional genetic modifiers of pain in people with PD is of high scientific and clinical interest and could open avenues for novel treatments. Here, we report the results of the first genome‐wide association study of pain in PD.

PD patients were recruited from the UK Parkinson's Pain Study, which included patients from the Tracking Parkinson's and the Oxford Parkinson's Disease Centre cohorts. The clinical assessment of pain in these patients has been previously reported.^1^ PD patients were stratified into 2 groups that represented individuals with no/low pain (McGill score < 3 and Visual Analog Scale severity <2) and high pain (McGill Score ≥ 3 and Visual Analog Scale severity ≥2).

DNA extracted from each sample was genotyped using either the Illumina Human ExomeCore‐12 v1.1 array, Illumina, Cambridge, UK (Tracking Parkinson's) or the InfiniumCoreExome‐24 v1.1, Illumina, Cambridge, UK (Oxford Parkinson's Disease Centre). Genotype data from both cohorts underwent the same conventional processing, quality control, and imputation procedures as described elsewhere.^3^

We performed a genome‐wide association study of 6,655,232 autosomal single nucleotide polymorphisms (SNPs) that compared a total of 898 patients with PD who were classed as suffering high levels of pain to 420 PD patients who were not experiencing pain. After including covariates for age, gender, and ancestry in the association analysis there was no evidence of genomic inflation attributable to population stratification (λ = 1.00).

This analysis identified 2 SNPs (rs11563208 and rs12465950) that were associated with pain in PD at genome‐wide significance (*P* = 1.45E‐09, odds ratio [OR] = 1.78, and *P* = 9.30E‐09, OR = 1.71, respectively; Fig. 1). The genotypes of these SNPs were strongly correlated (*r* ^2^ = 0.85) and are located at the gene encoding the human transient receptor potential cation channel, subfamily M, member 8 (*TRPM8*) on chromosome 2q37.1, with rs11563208 being a synonymous variant located within exon 22 and rs120465950 intronic. SNPs within *TRPM8* are established risk factors for migraine and headaches at genome‐wide significance.^4^ Using rs10166942 as a marker for the genetic association with migraine,^4^ conditional association analysis of pain in PD confirmed the strong association at rs11563208 (OR = 1.81, *P* = 4.2E‐08), supporting its independence to the genetic risk for migraine. An assessment of published genome‐wide association study data did not identify the lead SNPs at the *TRPM8* locus to be associated with any other pain phenotype.^5^

**Figure 1:**
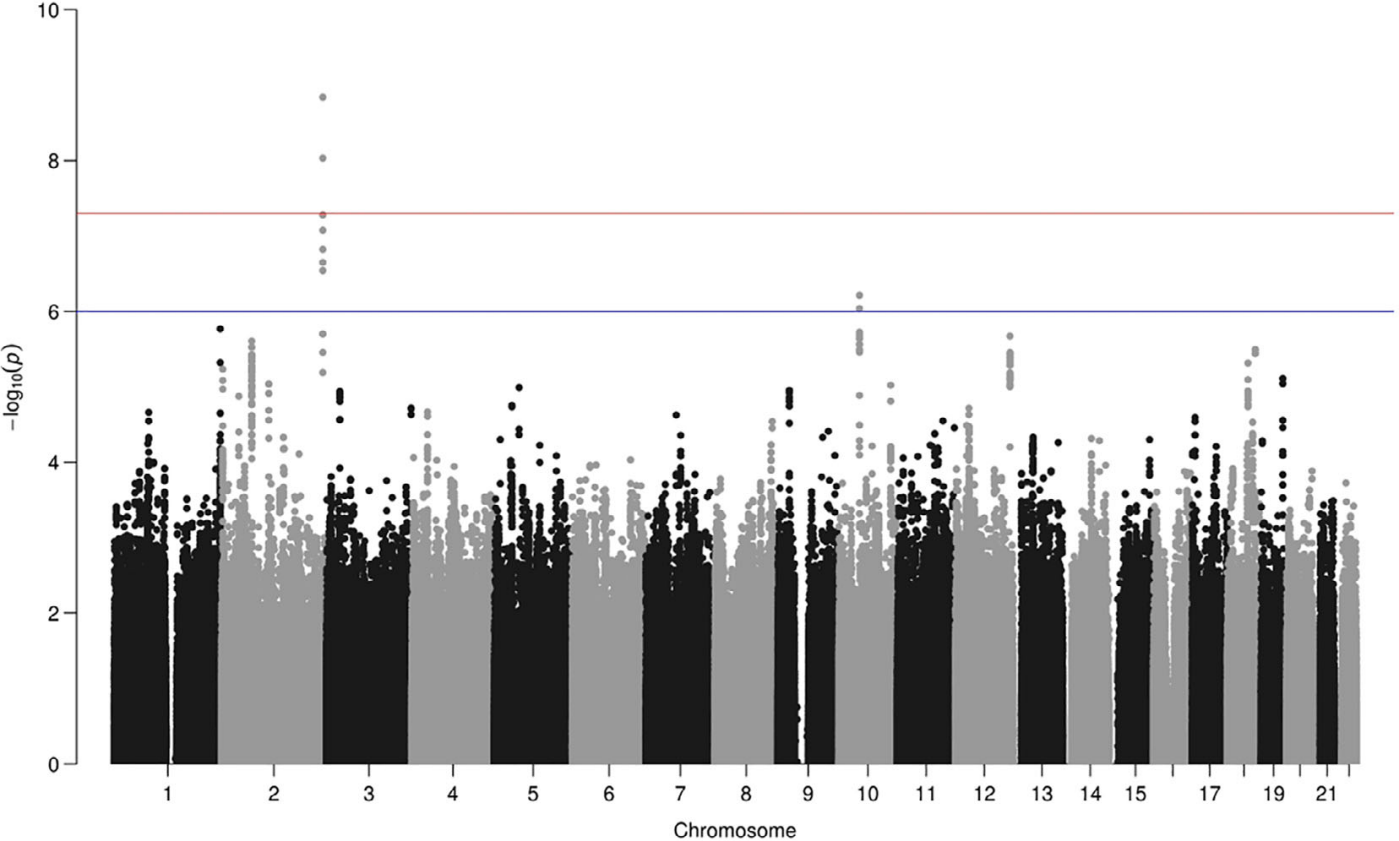
Manhattan plot of −log10 single nucleotide polymorphism *P* values from a meta‐analysis of high pain (n = 898) versus low pain (n = 420). Red and blue lines represent the thresholds for genome‐wide (P < 5E‐08) and suggestive (P < 1E‐06) significance, respectively.

TRPM8 has several reported functions, most notably as a cold/menthol thermoreceptor and is expressed in dorsal horn neurons.^6^ Genetic variants at this locus are also strongly associated with migraine susceptibility^4^; however, we note that our conditional analysis implies that these variants are independent of those associated with pain in PD in our analysis. This suggests the role of TRPM8 in PD pain may be different mechanistically to that of migraine. TRPM8 has been previously linked to chronic pain in animal models, and research is ongoing to identify compounds that effectively target TRPM8, with numerous antagonists being patented by pharmaceutical companies.^6^ Interestingly, cannabinoid ligands, compounds that have demonstrated efficacy as analgesic agents, have been shown to antagonize the TRPM8 receptor.^7^ Indeed, some authors have termed TRPM8 and other related Transient Receptor Potential channels as ionotropic cannabinoid receptors, suggesting that cannabinoids may be worth pursuing as treatments for PD pain.

In conclusion, we report the first genome‐wide significant evidence for association with pain susceptibility in PD, which implicates the gene *TRPM8*. The large body of evidence implicating this gene with migraine and chronic pain has already resulted in this gene being a pharmacologic target, and together with its known relationship with cannabinoids, opens novel therapeutic opportunities for this currently poorly managed symptom.

## Appendix: Members of the UK Parkinson's Pain Study

**Principal investigators**: C. Borland, A. Graham, S. Guptha, T. Ward, P. Worth, P. Sarda, W. Dwornik, G. Mamutse, J. Hindle, S. Jones, A. Church, R. Athey, D. Bathgate, N. Archibald, N. Pavese, D. Burn, U. Nath, M. Carson, R. Walker, T. Foltynie, A. Misbahuddin, A. Schrag, S. Sveinbjornsdottir, Y. Adenwala, B. Boothman, J. George, A. O'Callahan, M. O'Neill, J. Raw, M. Steiger, M. Wilson, K. Amar, S. Arianayagam, Z. Hemsley, H. Roberts, J. Stern, T. Andrews, D. Paviour, J. Frost, V. Lyell, T. Harrower, R. Sheridan, P. Critchley, L. Sugathapala, N. Bajaj, J. Sharma, C. Clarke, S. Ellis, K. Nithi, N. Kock, Z. Dhakam, C. Ruffman, P. Tomlinson, T. Quinell, S. Molloy, A. Whone, O. Bandmann, E. Capps, R. Bland.

## Research nurses

C. Downes, C. Vandor, J. Frost, D. Cooper, K. Dixon, R. Norton, M. Clarke, C. Hall, O. Olanrewaju, V. Hetherington, K. Blachford, S. Guptha, B. Bishop, D. Nesbitt, J. McEntee, F. Frenais, A. Hursey, E. Visentin, C. Edwards, J. Newman, r. james, E. Johnson, K. Ullyart, J. Brooke, I. Bataller, J. Rickett, T. Mahan, L. Craven, K. Powell, R. Humphries, B. Wilson, M. Trimmer, L. Whelan, A. McNichol, T. Fuller, L. Alderton, A. Henderson, P. Brown, V. Visentin, C. Parker, S. Large, H. Vanek, D. Wilson, A. Misbahudddin, F. Foster, S. Dube, E. Gunter, K. Amar, S. Dodds, G. Brown, G. Webster, P. Critchley, L. Foster, L. Dudgeon, K. Holmes, D. Nithi, K. K. Nithi, A. Donaldson, A. Dougherty, M. Misbahuddin, S. Atkinson, G. Carey, L. Catterall, D. Dellafera, C. Sunderland, A. Lyle, C. Sequeira, M. Hare, E. Ekins, K. Maitland, C. O'Reilly, M. Prabhakaran, P. Paterson, J. Connell, A. Pilcher, J. Birt, N. Vernon, T. McElwaine, M. Humphries, A. Lehn, T. Murphy, D. Critchley, M. Rolinsky, N. Temple, C. Brugaletta, S. Cable, D. Mills, S. Levy, E. Templeman, L. Wyatt, I. Massey, R. Innis, C. Ruffman, C. Schofield, E. Oughton, A. Davies, A. Lehn, M. Korley, N. Verstraelen, S. Morgan, W. Dwornik, J. Gilford, P. Aruldoss, A. Foster, C. Chaudhuri, L. Gethin, Y. Croucher, M. Williams, T. Roberts, R. Carson, R. Athey, V. Agarwal, P. Rachman, V. Ludley, T. Talbot, I. Inniss, R. Gentle, C. Hewitt, J. Stickley, E. White, K. Ward, S. Butler, S. Gallahawk, R. Fernandes, P. Zettergren, B. Tobin, K. Huyalt, U. Ullyart, E. Henderson, P. Forbes, M. Heywood, J. O'Brien, R. Rettergra, F. Bowring, S. Large, R. Roopun.

## Author Roles

(1) Research project: A. Conception, B. Organization, C. Execution; (2) Statistical Analysis: A. Design, B. Execution, C. Review and Critique; (3) Manuscript: A. Writing of the first draft, B. Review and Critique.

N.M.W.: 1A, 1B, 1C, 2A, 2B, 3A

L.H.: 1C, 2A, 2B, 3B

C.S.: 1C, 3B

C.W.: 1C, 3B

H.H.: 1C, 3B

M.L.:1C, 3B

C.C.:1C, 3B

K.R.C.: 1C, 3B

H.M.: 1C, 3B

M.T.H.: 1C, 3B

D.G.G.: 1C, 3B

C.K.: 1A, 1B, 1C, 2C, 3B

M.S.: 1A, 1B, 1C, 2A, 2B, 3A

## Financial disclosures of all authors (for the preceding 12 months):

N.M.W., L.H., C.S., C.W., and H.H. have no financial disclosures to report. M.L. is employed by a Parkinson's UK grant. C.C. has received meeting and consulting honoraria from Union Chimique Belge, Global Kinetics Corporation, Bial, Pfizer, Abidetex, EverPharma, and Abbvie as well as conference expenses from Bial. K.R.C. has received consultancy honoraria from AbbVie, UCB, Sunovion, Pfizer, Jazz Pharma, GKC, Bial, Cynapsus, Novartis, Lobsor, Stada, Medtronic, Zambon, Profile, and Sunovion; meeting honoraria from AbbVie, Britannia, UCB, Mundipharma, Zambon, Novartis, Boeringer Ingelheim Neuroderm, and Sunovion; grants from Britania Pharmaceuticals, AbbVie, UCB, GKC, Bial; and academic grants from European Union, Innovative Medicines Initiative EU, Horizon 2020, Parkinson's UK, National Institute for Health Research, Parkinson's disease Non Motor Group, EU (Horizon 2020), Kirby Laing Foundation, National Parkinson Foundation, and Medical Research Council. H.M. is employed by UCL. In the last 12 months he reports paid consultancy from Biogen, UCB, Abbvie, Denali, and Biohaven; lecture fees/honoraria from Biogen, UCB, C4X Discovery, GE‐Healthcare, Wellcome Trust, and the Movement Disorders Society; research grants from Parkinson's UK, Cure Parkinson's Trust, PSP Association, CBD Solutions, Drake Foundation, and Medical Research Council. Dr Morris is a coapplicant on a patent application related to C9ORF72—method for diagnosing a neurodegenerative disease (PCT/GB2012/052140). M.T.H. has undertaken consultancies for Biogen and Roche. The Oxford Discovery Cohort was funded by Parkinson's UK and is supported by NIHR and Dementias & Neurodegenerative Diseases Research Network. D.G.G. has received honoraria from Bial, UCB, and Merz Pharma. C.K. has received meeting and consulting honoraria from Bial and Abbvie as well as conference expenses from Bial and Merz. M.S. has received meeting honoraria from UCB as well as conference expenses from Bial and Abbvie.
